# Supplementary material for: Human drone interaction in delivery of medical supplies: A scoping review of experimental studies
Source: PLoS One. 2022 Apr 28;17(4):e0267664. doi: 10.1371/journal.pone.0267664 (PMC9049298; doi:10.1371/journal.pone.0267664)
Supplement: S1 Table — (DOCX) [file pone.0267664.s002.docx]

**S1 Table.** Search Strategy for Medline researcher 1.

| **Search number** | **Query** | **Search Details** | **Results** |
| --- | --- | --- | --- |
| #1 | drones OR UAV OR unmanned aerial vehicle OR UAS OR unmanned aerial systems OR unmanned aircraft system | drones[Title/Abstract] OR UAV[Title/Abstract] OR unmanned aerial vehicle[Title/Abstract] OR UAS[Title/Abstract] OR unmanned aerial systems[Title/Abstract] OR unmanned aircraft system[Title/Abstract] | 4,719 |
| #2 | **medical application OR medicine OR surgical application OR medical drones OR medicines OR vaccines** | **medical application[Title/Abstract] OR medicine[Title/Abstract] OR surgical application[Title/Abstract] OR medical drones[Title/Abstract] OR medicines[Title/Abstract] OR vaccines[Title/Abstract]** | 692,11 |
| #3 | **delivery OR support OR medical transport OR medical delivery OR delivery of healthcare** | **delivery[Title/Abstract] OR support[Title/Abstract] OR medical transport[Title/Abstract] OR medical delivery[Title/Abstract] OR delivery of healthcare[Title/Abstract]** | 1,507,601 |
| #4 | #2 AND #3 | (medical application[Title/Abstract] OR medicine[Title/Abstract] OR surgical application[Title/Abstract] OR medical drones[Title/Abstract] OR medicines[Title/Abstract] OR vaccines[Title/Abstract]) AND (delivery[Title/Abstract] OR support[Title/Abstract] OR medical transport[Title/Abstract] OR medical delivery[Title/Abstract] OR delivery of healthcare[Title/Abstract]) | 62,090 |
| #5 | #1 AND #4 | (drones[Title/Abstract] OR UAV[Title/Abstract] OR unmanned aerial vehicle[Title/Abstract] OR UAS[Title/Abstract] OR unmanned aerial systems[Title/Abstract] OR unmanned aircraft system[Title/Abstract]) AND ((medical application[Title/Abstract] OR medicine[Title/Abstract] OR surgical application[Title/Abstract] OR medical drones[Title/Abstract] OR medicines[Title/Abstract] OR vaccines[Title/Abstract]) AND (delivery[Title/Abstract] OR support[Title/Abstract] OR medical transport[Title/Abstract] OR medical delivery[Title/Abstract] OR delivery of healthcare[Title/Abstract])) | 19 |
| #6 |  | **user centered model OR user centered design framework [all fields]** | 654 |
| #5 AND #6 |  | **((drones[Title/Abstract] OR UAV[Title/Abstract] OR unmanned aerial vehicle[Title/Abstract] OR UAS[Title/Abstract] OR unmanned aerial systems[Title/Abstract] OR unmanned aircraft system[Title/Abstract]) AND ((medical application[Title/Abstract] OR medicine[Title/Abstract] OR surgical application[Title/Abstract] OR medical drones[Title/Abstract] OR medicines[Title/Abstract] OR vaccines[Title/Abstract]) AND (delivery[Title/Abstract] OR support[Title/Abstract] OR medical transport[Title/Abstract] OR medical delivery[Title/Abstract] OR delivery of healthcare[Title/Abstract]))) AND (user centered model OR user centered design framework)** | 0 |
| #7 | human drone interaction |  | 1 |
| #2 AND #3 AND #7 |  | **((delivery OR support OR medical transport OR medical delivery OR delivery of healthcare) AND (medical application OR medicine OR surgical application OR medical drones OR medicines OR vaccines)) AND (human drone interaction)** | 4 |
| #8 |  | **COVID OR SARS-CoV2 OR corona** | 116,258 |
| #5 AND #6 AND #8 |  | **((drones[Title/Abstract] OR UAV[Title/Abstract] OR unmanned aerial vehicle[Title/Abstract] OR UAS[Title/Abstract] OR unmanned aerial systems[Title/Abstract] OR unmanned aircraft system[Title/Abstract]) AND ((medical application[Title/Abstract] OR medicine[Title/Abstract] OR surgical application[Title/Abstract] OR medical drones[Title/Abstract] OR medicines[Title/Abstract] OR vaccines[Title/Abstract]) AND (delivery[Title/Abstract] OR support[Title/Abstract] OR medical transport[Title/Abstract] OR medical delivery[Title/Abstract] OR delivery of healthcare[Title/Abstract]))) AND (COVID[Title/Abstract] OR corona[Title/Abstract] OR SARS-CoV2[Title/Abstract])** | 0 |
