# Supplementary material for: Human drone interaction in delivery of medical supplies: A scoping review of experimental studies
Source: PLoS One. 2022 Apr 28;17(4):e0267664. doi: 10.1371/journal.pone.0267664 (PMC9049298; doi:10.1371/journal.pone.0267664)
Supplement: S2 Table — (DOCX) [file pone.0267664.s003.docx]

**S2 Table.** Search Strategy for Medline researcher 2.

| **Search number** | **Query** | **Search Details** | **Results** |
| --- | --- | --- | --- |
| #1 | human drone interaction | human drone interaction[Title/Abstract] | 1 |
| #2 | drone OR unmanned aerial vehicle OR drone aircraft OR unmanned drone | drone[Title/Abstract] OR unmanned aerial vehicle[Title/Abstract] OR ((drone[All Fields] OR drones[All Fields]) AND aircraft[Title/Abstract]) OR (unmanned[All Fields] AND drone[Title/Abstract]) | 1,738 |
| #3 | medical delivery OR medical transport | medical delivery[Title/Abstract] OR medical transport[Title/Abstract] | 613 |
| #4 | medicine OR medical device OR nurse OR nursing OR home care OR professional care | medicine[Title/Abstract] OR medical device[Title/Abstract] OR nurse[Title/Abstract] OR nursing[Title/Abstract] OR home care[Title/Abstract] OR professional care[Title/Abstract] | 925,885 |
| #5 | #1 AND #2 AND #3 AND #4 | human drone interaction[Title/Abstract] AND (drone[Title/Abstract] OR unmanned aerial vehicle[Title/Abstract] OR ((drone[All Fields] OR drones[All Fields]) AND aircraft[Title/Abstract]) OR (unmanned[All Fields] AND drone[Title/Abstract])) AND (medical delivery[Title/Abstract] OR medical transport[Title/Abstract]) AND (medicine[Title/Abstract] OR medical device[Title/Abstract] OR nurse[Title/Abstract] OR nursing[Title/Abstract] OR home care[Title/Abstract] OR professional care[Title/Abstract]) | 0 |
| #6 | #2 AND #3 AND #4 | (drone[Title/Abstract] OR unmanned aerial vehicle[Title/Abstract] OR ((drone[All Fields] OR drones[All Fields]) AND aircraft[Title/Abstract]) OR (unmanned[All Fields] AND drone[Title/Abstract])) AND (medical delivery[Title/Abstract] OR medical transport[Title/Abstract]) AND (medicine[Title/Abstract] OR medical device[Title/Abstract] OR nurse[Title/Abstract] OR nursing[Title/Abstract] OR home care[Title/Abstract] OR professional care[Title/Abstract]) | 2 |
| #7 | #2 AND #3 | (drone[Title/Abstract] OR unmanned aerial vehicle[Title/Abstract] OR ((drone[All Fields] OR drones[All Fields]) AND aircraft[Title/Abstract]) OR (unmanned[All Fields] AND drone[Title/Abstract])) AND (medical delivery[Title/Abstract] OR medical transport[Title/Abstract]) | 2 |
