# Supplementary material for: Human drone interaction in delivery of medical supplies: A scoping review of experimental studies
Source: PLoS One. 2022 Apr 28;17(4):e0267664. doi: 10.1371/journal.pone.0267664 (PMC9049298; doi:10.1371/journal.pone.0267664)
Supplement: S3 Table — (DOCX) [file pone.0267664.s004.docx]

**S3 Table.** Search Strategy for CINAHL researcher 1.

| **Search number** | **Query** | **Search Details** | **Results** |
| --- | --- | --- | --- |
| #1 | drones OR UAV OR unmanned aerial vehicle OR UAS OR unmanned aerial systems OR unmanned aircraft system | drones[Title/Abstract] OR UAV[Title/Abstract] OR unmanned aerial vehicle[Title/Abstract] OR UAS[Title/Abstract] OR unmanned aerial systems[Title/Abstract] OR unmanned aircraft system[Title/Abstract] | 390 |
| #2 | **medical application OR medicine OR surgical application OR medical drones OR medicines OR vaccines** | **medical application[Title/Abstract] OR medicine[Title/Abstract] OR surgical application[Title/Abstract] OR medical drones[Title/Abstract] OR medicines[Title/Abstract] OR vaccines[Title/Abstract]** | 68,766 |
| #3 | **delivery OR support OR medical transport OR medical delivery OR delivery of healthcare** | **delivery[Title/Abstract] OR support[Title/Abstract] OR medical transport[Title/Abstract] OR medical delivery[Title/Abstract] OR delivery of healthcare[Title/Abstract]** | 192,852 |
| #4 | #2 AND #3 | (medical application[Title/Abstract] OR medicine[Title/Abstract] OR surgical application[Title/Abstract] OR medical drones[Title/Abstract] OR medicines[Title/Abstract] OR vaccines[Title/Abstract]) AND (delivery[Title/Abstract] OR support[Title/Abstract] OR medical transport[Title/Abstract] OR medical delivery[Title/Abstract] OR delivery of healthcare[Title/Abstract]) | 7,159 |
| #5 | #1 AND #4 | (drones[Title/Abstract] OR UAV[Title/Abstract] OR unmanned aerial vehicle[Title/Abstract] OR UAS[Title/Abstract] OR unmanned aerial systems[Title/Abstract] OR unmanned aircraft system[Title/Abstract]) AND ((medical application[Title/Abstract] OR medicine[Title/Abstract] OR surgical application[Title/Abstract] OR medical drones[Title/Abstract] OR medicines[Title/Abstract] OR vaccines[Title/Abstract]) AND (delivery[Title/Abstract] OR support[Title/Abstract] OR medical transport[Title/Abstract] OR medical delivery[Title/Abstract] OR delivery of healthcare[Title/Abstract])) | 10 |
