# Supplementary material for: Human drone interaction in delivery of medical supplies: A scoping review of experimental studies
Source: PLoS One. 2022 Apr 28;17(4):e0267664. doi: 10.1371/journal.pone.0267664 (PMC9049298; doi:10.1371/journal.pone.0267664)
Supplement: Attachment — Submitted filename: responses_to_reviewers_PONE-D-21-23464_final.docx [file pone.0267664.s007.docx]

**Responses to reviewers**

Manuscript Number: PONE-D-21-23464

Human drone interaction in delivery of medical supplies: a scoping review of experimental studies

PLOS ONE

Dear Rafael Santos Santana,

Thank you very much for the opportunity to submit a revised version of our manuscript. We are very thankful for the constructive comments provided by you and the reviewers. In the following, we address all reviewers´ comments in a point-by-point fashion and hope the manuscript is now ready for publication in PLOS ONE. Changed paragraphs were marked in blue color throughout the manuscript.

Thank you very much.

Yours sincerely,

Franziska Stephan (corresponding author), on behalf of all co-authors

**Editor´s comments**

**When submitting your revision, we need you to address these additional requirements.**

**1. Please ensure that your manuscript meets PLOS ONE's style requirements, including those for file naming. The PLOS ONE style templates can be found at**

[**https://journals.plos.org/plosone/s/file?id=wjVg/PLOSOne_formatting_sample_main_body.pdf**](https://journals.plos.org/plosone/s/file?id=wjVg/PLOSOne_formatting_sample_main_body.pdf) **and**

[**https://journals.plos.org/plosone/s/file?id=ba62/PLOSOne_formatting_sample_title_authors_affiliations.pdf**](https://journals.plos.org/plosone/s/file?id=ba62/PLOSOne_formatting_sample_title_authors_affiliations.pdf)**”**

**Response:** We have checked the style templates for the title page and manuscript. We included the current address from DP due to professional changes. We have noticed that one affiliation was missing, which we have now added. Please refer to title page for changes.

**2. Thank you for stating the following in the funding Section of your manuscript:**

**“FKZ03 funding code: 03COV25E” We would like to thank the ADApp-Team for exchange and discussion of human drone interaction. A special thank goes to Nicole Reinsperger (NR) for their help during systematic literature research. We also would like to thank Jamie Smith for editing the manuscript.**

**We note that you have provided funding information that is not currently declared in your Funding Statement. However, funding information should not appear in the Acknowledgments section or other areas of your manuscript. We will only publish funding information present in the Funding Statement section of the online submission form.**

**Please remove any funding-related text from the manuscript and let us know how you would like to update your Funding Statement. Currently, your Funding Statement reads as follows:**

**“This research was funded by the Federal Ministry of Education and Research Germany through the “TDG - Translational region of Digital Health Care” project [03COV25E]. PJ receives this funding. The funders had no role in study design, data collection and analysis, decision to publish, or preparation of the manuscript.”**

**Please include your amended statements within your cover letter; we will change the online submission form on your behalf.**

**Response:** We have checked the manuscript for funding information. We deleted the part of funding information (p. 6). There is nothing that we would like to be changed in the Funding Statement in the online submission form.

**3. Please include your full ethics statement in the ‘Methods’ section of your manuscript file. In your statement, please include the full name of the IRB or ethics committee who approved or waived your study, as well as whether or not you obtained informed written or verbal consent. If consent was waived for your study, please include this information in your statement as well.**

**Response:** Thank you for your comment. We included our full ethics statement in the Methods section.

*The scoping review was included in the ADApp study. The ADApp study was approved by the Ethics Committee of the Martin-Luther-University Halle-Wittenberg (protocol code 2021-069 and date of approval May 6, 2021). All participants gave written informed consent.*

**4. "We note that this manuscript is a systematic review or meta-analysis; our author guidelines therefore require that you use PRISMA guidance to help improve reporting quality of this type of study. Please upload copies of the completed PRISMA checklist as Supporting Information with a file name “PRISMA checklist”."**

**Response:** Thank you for your comment. We have renewed the PRISMA checklist according to the PRISMA extension for Scoping Reviews (PRISMA-ScR) and uploaded the checklist as Supporting Information named “Appendix S1_PRISMA-ScR checklist_PONE-D-21-23464”.

**Reviewer 1**

**1. Is the manuscript technically sound, and do the data support the conclusions?

Reviewer #1: Partly**

**2. Has the statistical analysis been performed appropriately and rigorously?**

**Reviewer #1: N/A**

**3. Have the authors made all data underlying the findings in their manuscript fully available?

Reviewer #1: Yes**

**4. Is the manuscript presented in an intelligible fashion and written in standard English?

Reviewer #1: Yes**

**5. Review Comments to the Author**

**The theme is exciting and has an interesting and current approach. I believe that some adjustments are needed to bring more fluidity to the work and the reader's attention.**

**Response:** Thank you very much for your review and your relevant suggestions. We address all your comments in a point-by-point fashion.

**The introduction is well written, but it is worth reviewing/restructuring considering a macroscopic perspective from the use of drones to the key idea of the study ("medical delivery processes").**

**Response:** Thank you very much for you careful considerations. We now restructured the introduction from a macroscopic perspective to a more fine-grained perspective. Please refer to the whole introduction for a complete view.

*“A recent population survey provides information about the attitude, the willingness of use, expectations, and fears of the German population towards delivery drones and air cabs [9]. Overall, the results showed that the majority of the German population is skeptical about the delivery of consumer goods by drone. However, exceptions to this are emergency situations, e.g., the delivery of drugs or for the transportation of injured persons to hospital. The authors suggested a greater consideration of social ideas, doubts, and requirements in the design and further development of drone technology. This requires the involvement of users to improve the effectiveness of the utilization of medical delivery to ensure that the process and technique remain patient-focused [9,10]. In this vein, ethical challenges are related with humanitarian innovations, including drones. An international review identified three major trends in ethical considerations [11]. They found issues related to harm (i.e., focus on ensuring physical safety, environmental impacts and benefits), justice (i.e., cost-effectiveness, equitable access and stakeholder responsibility), and respect (i.e., technical aspects of information security, considerations of privacy, active community engagement). These considerations imply a higher involvement of users and stakeholders in future research to improve harm-benefit trade-offs, upholding justice, and respect autonomy. To date, reviews often focus on how drones are used for healthcare and its potentials [11–13]. For example, Hievert et al. [12] identified eight healthcare and health-related applications for drones. Drone delivered medical supplies and treatments was the most common identified application followed by environmental monitoring and using drones to deliver automated defibrillators (AEDs) indicating the importance of drones in improving access to health services. The review found out that all studies on drones are at a pilot stage that have not been implemented or adopted in healthcare settings. The identified studies focused on flight path and payload regulations, but there was limited attention to how patients and communities interact with drones. Rejeb et al. [13] identified three main barriers to humanitarian drones in technological, organizational, and environmental (regulatory) factors, called TOE barriers. Among other things, user acceptance is one organizational barrier to drone adoption in humanitarian logistics. The authors recommend applying technology adoption theories as well as using case studies, interviews, or surveys to understand success and failure factors of using drones within humanitarian organizations. Hence, analysis of human drone interaction is required for understanding users’ needs and requirements in dealing and interaction with drones. The role of users, the involvement of users in drone delivery process and the handover of medical devices or urgently needed medicines are important factors in investigating and improving user acceptance. In the end, it is the user who will have to use the drone in everyday life and get along with it. Non-user-centered technologies will fail to be implemented as long as usefulness and usability are not a focus of research. As long as this is the case, people´s fears and concerns about drones will be the main obstacles to implementation although drones have a great potential in medical care and supply. Within this healthcare supply context, the present paper is interested in identifying human drone interaction in real life scenarios that have received less attention in the ongoing debate. More specifically, the objective is to assess how human drone interaction in medical transport of medicines and devices in experimental studies is discussed in the academic literature.”* (p. 5-6)

**In the methodology, clarify what the asterisks in table 1 refer to.**

**Response:** We included a legend below the table. The asterisks display truncations.

**Clarify what "Source types included reviews" is (line 115) given that "This review aims to assess only experimental studies because they provide evidence to support the use, acceptance and effectiveness of drones in medical delivery processes" (129-131).**

**Response:** Thank you for your comment. We now clarified why we included reviews in the initial search:

*“Initially, both reviews and empirical studies were included in the search. Although the present scoping review aims to assess only empirical studies, reviews were initially included for identifying studies that may have been missed in the database search but were included in reference lists of reviews.”* (p. 8)

**I suggest better describing "search update" (166) (would it be: "references of identified publications were searched" in lines 28/29 of the abstract?).**

**Response:** Thank you, we agree that “search update” is not specific enough. We now rephrased the whole section for a better understanding:

*“The research was conducted in May 2021. The deadline after which no more studies would have been included in the analyses was the 31^th^ May 2021. In the initial search, 83 results were retrieved: 71 from PubMed and 22 from CINAHL. After removing duplicates, 58 results were included in the initial screening. Titles and abstracts from these 58 studies were assessed. 41 studies were excluded because they either did not have empirical data, a drone or medical reference, or included drones used in military and warfare. The remaining 17 results were then screened for their references according to the research question. Seven reviews, two perspectives, and eight studies were screened according to references. After reference screening, all reviews and perspectives were excluded from further analyses. One study was added with regard to the delivery process of medicine, resulting in four studies, and two studies for the delivery of medical device process, resulting in seven studies. Next, the identified studies were full-text screened. However, all four studies associated with the delivery of medicines were subsequently excluded because they did not involve human subjects or the delivery of organ samples. For the defibrillator delivery process, three studies were excluded because the study did not include empirical data or human subjects.* ***Figure 1*** *depicts the contribution of all exclusion criteria. Overall, four studies were selected for the systematic synthesis and are summarized in Table 3. See* ***Figure 2*** *for the systematic search and study selection process. Please refer to additional file S6 for an overview of excluded studies and the reason of exclusion.”* (p. 12-13)

***Figure 1.*** *Frequency of exclusion criteria.*

**In the results, the section "Human drone interaction in AED Delivery" could be structured in a more fluid and interesting way for the reader. It is a fundamental (and very interesting) part of the work, but it can be presented in a more objective way and even with the use of comparative tables.**

**Response:** Thank you very much for the helpful comment. We integrated a table (Table 4) which shows a summary of user experience, user feedbacks, and practical implication in the Result section, which was then discussed in the Discussion section. Moreover, we restructured the section “Human drone interaction in AED delivery”. Please refer to the whole section for a complete view.

**Table 4.** User experience, feedback, and practical implications.

|  | **problems** | **users´ concerns** | **users´ perceived support** | **suggestions of users** | **advantages from the researchers´ point of view** | **practical implications** |
| --- | --- | --- | --- | --- | --- | --- |
| (1) process / landing |  | uncertainty of arrival time [19,20] |  | noise emitting or something that alerts drone arrival [20] |  | test flights at day and night, using headlights, warning sensors, reflective tapes or sound |
|  |  | flight at night [20] |  | reflective tapes or lights for marking location of arriving [20] | in case of landing: warning sensors, lights, and sound to attract attention [17] |  |
|  |  | direct physical contact with drone: drone too close or propeller starts when approaching drone [18–20] |  | upward inclined or retractable propeller [18] | use a winch to avoid contact with drone [18] | release with winch over landing |
|  |  | landing in crowd areas [20] |  |  |  |  |
|  |  | issues with bystanders [20] |  |  |  |  |
|  |  | poor weather [20] |  |  |  | investigating complementary and interoperability of drones with other technologies |
|  |  | drone´s ability to find right location [20] |  |  |  | using applications with GPS tracking |
|  | using mobile phones [18] |  |  |  |  | educational training |
| (2) handover |  | finding AED fast enough [18,20] | drone´s hovering to find right location [18] | headlights in order to mark location of release [18] | live video streaming for facilitation of dispatcher orientation [18] | documentation of handover via live video streaming or app-based confirmation |
|  |  |  | red AED bag for easier identification on ground [18,20] |  |  |  |
|  | difficulty in removing AED from drone [20]  and  difficulties in handling AED despite dispatcher support [18] |  |  | colored Velcro tapes and additional labels with removing instructions [20] | suggestion of more intuitive location, e.g., on the top of drone [17] | using applications with instructions, e.g. via video |
| (3) communication |  | anxiety / uncertainty about direction or timing of drone´s arrival/landing [19,20] | interaction with dispatcher gave sense of security [18] | clearer instructions from dispatcher about appearance, look and sound of drone, direction, and landing [20] |  | clear and short instructions, education of professionals (e.g., dispatcher, pharmacists) |
|  | long sentences affect situation in negative way [18] |  |  |  | short encouraging sentences  observed positive  effect on compressions [18] |  |

**In the discussion I believe that there can be a greater approach on: 1. the articles were only developed countries (although, as presented in the article, there is use of drones in several countries); 2. equipment only (all four studies addressed an out-of-hospital cardiac arrest; 3. three studies used drones from DJI), little variation; 4. focus on rural areas. Add limitations that could potentially influence the results obtained.**

**Response:** Thank you very much for your helpful consideration. We included a new section called “Practical and Theoretical Implications” in which we discuss the results in a broader context and in the light of previous reviews and theories. Please refer to the whole Discussion section for a complete view. We also integrated a section with limitations of the study:

***Limitations***

*Although the scoping review was supported by steps including refinement of the protocol through team discussion, blinded searching, and selection of articles by two researchers, several limitations have to be mentioned. First, the present study did not include gray literature. Although the search included two relevant academic databases with peer-review journal articles, gray literature might also yield additional insights. Thus, it is possible that studies with human drone interaction focus have been overlooked. Second, the search protocol was based on a combination of keywords that may not capture all relevant studies. Third, the theoretical and practical implications derived from this scoping review need to be tested and validated with empirical research methods. Forth, we adopted a relatively narrow approach to the context by including supply of medicines and medical devices. Expanding the context might contribute to more hits according to human drone interaction settings. Nevertheless, the strength of this narrow context is to provide an insight in specific domains and its potential.* (p. 31)

**Reviewer 2**

**1. Is the manuscript technically sound, and do the data support the conclusions?

Reviewer #2: Partly**

**2. Has the statistical analysis been performed appropriately and rigorously?**

**Reviewer #2: N/A**

**3. Have the authors made all data underlying the findings in their manuscript fully available?

Reviewer #2: No**

**4. Is the manuscript presented in an intelligible fashion and written in standard English?

Reviewer #2: Yes**

**5. Review Comments to the Author**

**Thank you for allowing me the opportunity to read your manuscript and provide feedback on your work. I respectfully submit the comments to help improve your article.**

**1. Summary of the research and overall impression

The work addresses the use of drones with application in public health. This is a relatively new topic and about which, in the coming years, growth in the literature should be observed. The methodological choice of a scoping review is pertinent and adequate, although there is a need to add more information to improve the clarity of the work and allow better compression. I have pointed out some critical issues that I would like the authors to elucidate. I hope the comments can be useful to improve the quality of the work. Thank you again for allowing me to review your work.**

**Response:** Thank you very much for your review and your valuable considerations. We address all your comments in a point-by-point fashion.

**2. Major issues

Introduction

2.1. Line 78: in a non-exhaustive search in Pubmed, I found a scoping review that analyzed the use of drones in humanitarian contexts (DOI: 10.1007/s11948-021-00327-4). Authors should review the phrase that refers to the non-existence of scoping review on the subject and analyze the relevance of including the referred work in the review.**

**Response:** Thank you very much for you careful considerations. We now rephrased the introduction and included your suggested review. We also did a search update and found another review, which we included in the introduction, too. We could not find these reviews because our systematic research was conducted in May 2021 and we submitted our manuscript in July 2021 and resubmitted it in August 2021. The review of Wang et al. (2021) was published in August 2021 and the review of Rejeb et al. (2021) in July 2021. However, all reviews have in common that they investigate the potential and benefit of drones and its use in healthcare. But to our knowledge, the focus on human drone interaction was not explicitly addressed. Successful implementation of any health system change relies on a complex set of factors. One factor is the human drone interaction and the acceptance of users (e.g., patients, nurses) because they will be the one who have to implement and use the system in their routines and daily life’s. Please refer to the whole introduction for a complete view.

*“A recent population survey provides information about the attitude, the willingness of use, expectations, and fears of the German population towards delivery drones and air cabs [11]. Overall, the results showed that the majority of the German population is skeptical about the delivery of consumer goods by drone. However, exceptions to this are emergency situations, e.g., the delivery of drugs or for the transportation of injured persons to hospital. The authors suggested a greater consideration of social ideas, doubts, and requirements in the design and further development of drone technology. This requires the involvement of users to improve the effectiveness of the utilization of medical delivery to ensure that the process and technique remain patient-focused [9,10]. In this vein, ethical challenges are related with humanitarian innovations, including drones. An international review identified three major trends in ethical considerations [11]. They found issues related to harm (i.e., focus on ensuring physical safety, environmental impacts and benefits), justice (i.e., cost-effectiveness, equitable access and stakeholder responsibility), and respect (i.e., technical aspects of information security, considerations of privacy, active community engagement). These considerations imply a higher involvement of users and stakeholders in future research to improve harm-benefit trade-offs, upholding justice, and respect autonomy. To date, reviews often focus on how drones are used for healthcare and its potentials [11–13]. For example, Hievert et al. [12] identified eight healthcare and health-related applications for drones. Drone delivered medical supplies and treatments was the most common identified application followed by environmental monitoring and using drones to deliver automated defibrillators (AEDs) indicating the importance of drones in improving access to health services. The review found out that all studies on drones are at a pilot stage that have not been implemented or adopted in healthcare settings. The identified studies focused on flight path and payload regulations, but there was limited attention to how patients and communities interact with drones. Rejeb et al. [13] identified three main barriers to humanitarian drones in technological, organizational, and environmental (regulatory) factors, called TOE barriers. Among other things, user acceptance is one organizational barrier to drone adoption in humanitarian logistics. The authors recommend applying technology adoption theories as well as using case studies, interviews, or surveys to understand success and failure factors of using drones within humanitarian organizations. Hence, analysis of human drone interaction is required for understanding users’ needs and requirements in dealing and interaction with drones. The role of users, the involvement of users in drone delivery process and the handover of medical devices or urgently needed medicines are important factors in investigating and improving user acceptance. In the end, it is the user who will have to use the drone in everyday life and get along with it. Non-user-centered technologies will fail to be implemented as long as usefulness and usability are not a focus of research. As long as this is the case, people´s fears and concerns about drones will be the main obstacles to implementation although drones have a great potential in medical care and supply. Within this healthcare supply context, the present paper is interested in identifying human drone interaction in real life scenarios that have received less attention in the ongoing debate. More specifically, the objective is to assess how human drone interaction in medical transport of medicines and devices in experimental studies is discussed in the academic literature.”* (p. 5-6)

**2.2. Lines 86-99: authors are requested to include more details about population and context, in line with the mnemonic PCC (population, concept, and context) found in the JBI 2020 manual (**[**https://doi.org/**](https://doi.org/) **10.46658/JBIMES-20-12).**

**Response:** Thank you very much for your comment. We rephrased the introduction in a hopefully clearer way and include more details about the PCC. Our context is healthcare supply (especially the medical supply and medical device supply), focusing on human drone interaction in real life scenarios in experimental studies. Because the focus is on the concept and context, the population is widely defined as “users”, i.e., all who are requested to interact with the drone in the context of medical supply or medical device supply. See also the new Table 2. The changed section now reads as following:

*“Within this healthcare supply context, the present paper is interested in identifying human drone interaction in real life scenarios that have received less attention in the ongoing debate. More specifically, the objective is to assess how human drone interaction in medical transport of medicines and devices in experimental studies is discussed in the academic literature. The scoping review addresses the following question:*

*How does the human drone interaction take place in the delivery of medication or medical device (defibrillator)?*

*The current review is especially interested in the role of users (i.e., all persons who are requested to interact with the drone, such as patients, nurses, bystanders, victims, or voluntaries independent of age or disease) in the delivery practice and their experiences, in the procedure of drone delivery, the handover, and the health-related outcomes in healthcare supply (medication supply) and health-related applications (supply with medical devices). In this work, therefore, the central notions are “human drone interaction”, “delivery”, and “medication”, or “medical device”.”* (p. 6-7)

**Table 2.** Summary of review criteria.

| **Review criteria** | **Description** |
| --- | --- |
| Publication year | No temporal restriction was applied |
| Publication language | No language restriction was applied |
| Publication type | *Inclusion criteria:* Only journal articles with empirical methods (e.g., interview, focus groups, questionnaires); reviews were included only for reference checking  *Exclusion criteria:* articles without empirical design (e.g., reports, websites) |
| Concept | *Inclusion criteria:* human drone interaction (e.g., acceptance, usability, user experience, user-centeredness) in real life scenarios |
| Context | *Inclusion criteria:* supply of medicines or medical devices  *Exclusion criteria:* no healthcare context (e.g., military or warfare use) |
| Population | *Inclusion criteria:* human subjects (independent of age, disease, background)  *Exclusion criteria:* simulation studies without human subjects |

**Methods

2.3. Line 103: the authors cite PRISMA 2020 as a method for systematic search. The text should be reviewed since PRISMA is a guideline for reporting systematic reviews and not a guide for their preparation. Due to my language limitation in German, I was unable to evaluate the full text of citation #13 which references a guide for conducting scope reviews, based on the methodology returned by JBI. It should be noted that the official JBI 2020 guidelines for conducting scoping reviews can be accessed at** [**https://doi.org/10.46658/JBIMES-20-12**](https://doi.org/10.46658/JBIMES-20-12) **and should preferably be used in references to the method for preparing a scoping review.**

**Response:** Thank you for your comment. We agree that we failed to describe the methodological guidelines for systematic literature research and reporting favorably. We rephrased the paragraph that now reads as follows:

*“To identify research gaps and to examine the range and extent of research activity in human drone interaction (user´s role, experience, and acceptance), we performed a scoping review [14]. Following the systematic literature research (SLR), the present scoping review followed the stages (1) identifying the research question, (2) identifying relevant studies, (3) study selection, (4) charting the data, and (5) collating, summarizing, reporting the results* [14]. *For improving the rigor, comprehensiveness, and credibility of reporting methods and results of the present scoping review, we followed the PRISMA-ScR Checklist (Preferred Reporting Items for Systematic reviews and Meta-Analyses extension for Scoping Reviews) and JBI (Joanna Briggs Institute) guidelines [15]. The PRISMA-ScR checklist is provided in the additional file S1.”* (p. 7)

**2.4. Line 105: the authors cite the preprint of the manuscript containing the PRISMA update for systematic reviews (DOI: 10.31222/osf.io/v7gm2). However, the article has already been published in several journals (https://www.equator-network.org/reporting-guidelines/prisma/). Considering the existence of the PRISMA Extension for Scoping Reviews (**[**https://doi.org/10.7326/M18-0850**](https://doi.org/10.7326/M18-0850)**), it is requested that this instrument be used instead of PRISMA for systematic reviews.**

**Response:** Thank you for your very important considerations. We now use the PRISMA-ScR, adapted it in the method section (p. 7, see previous comment), checked it throughout the manuscript and changed the table in additional file S1. Please refer to the whole manuscript for changes regarding PRISMA-ScR.

**2.5. Line 146-147: the manuscript reports that the final data extraction was performed by one of the authors. There is no mention of whether a second author reviewed the extraction and whether there was an assessment of consensus among the authors. Please submit more information.**

**Response:** You are right that we missed reporting that a second author reviewed the extraction. We hence included this information:

*“Two researchers (FS, NR) independently performed the searches and screened all studies with regard to the search terms in title and abstract as well as for inclusion criteria in order to exclude studies that were not relevant to the review. To increase consistency among researchers, all researchers screened the same studies. If the relevance of a study was unclear from abstract, the full text was screened. It was set a deadline, after which it was agreed that no more studies would be included in the analyses. Researcher 1 identified 19 publications via MEDLINE and ten via EBESCO. Researcher 2 found two studies via MEDLINE and ten via EBESCO. In the event of disagreement in the selection process, a discussion between these two researchers was carried out until the researchers reached consensus. All studies that met inclusion criteria proceeded to data extraction. Final data extraction involving full-text screening of each included study. Two authors (FS and DP) who independently reviewed the full text and made decision on whether to include or exclude the study based on the aims of this scoping review. Disagreements were resolved by consensus.* *A data-charting form was jointly developed by the two researchers (see additional file S6). The researchers independently charted the data and discussed the results. The form was continuously updated within an iterative process. The researchers abstracted data of all studies on article characteristics (publication year, country of origin, publication type), case numbers, and contextual factors (study design and research question). For final data extraction, data were abstracted on results (e.g., outcomes of interest), critical features (e.g., drone involvement in the process, drone request, role of users, type of user group (trained or untrained), handover), and participant characteristics, and were then compared with inclusion criteria. Since a scoping review does not conduct quality appraisal, critical appraisal was omitted [14]. The researchers grouped the studies according to inclusion and exclusion criteria. In case a study met the inclusion criteria, results and critical features were summarized in a table (additional file S6).“ (p. 11-12)*

**2.6. The authors do not mention whether a search for gray literature was performed. Please justify if it has not been performed.**

**Response:** A search for gray literature was not performed. We included this information in the “Search Strategy” section. The targeted search for systematic and methodical works had priority in the present study. Hence, this decision was made due to the fact that the initial desk research allowed us to conclude that, although there were a large number of reports on the topic, they often lacked a scientific foundation. Moreover, in these searches we already encountered the references to substantive and methodologically sound studies, therefore the integration of narrative reports was dismissed. Nevertheless, we included the information not searching for gray literature in the Limitation section.

*“A search for gray literature was not performed because the targeted search for systematic and methodological works had priority in the present study. This decision was made due to the fact that initial desk research revealed a large number of reports on the topic which, however, often a lacked scientific foundation.”* (p. 8)

**2.7. The authors did not report if a one priori protocol for the scoping review was created. Scoping review protocols can be registered on platforms such as “Open Science Framework” or “Figshare”, or even published in journals. If it has been created, it is requested that the code and a source be included to access it.**

**Response:** Thank you very much for your comment. You are right that we should register the protocol. No registration was made since the scoping review is embedded in a mixed-methods study design and is intended to provide an initial overview of the research landscape. Above all, it became clear that well-founded results were not available in the mentioned time in relation to the overall objective of the “ADApp”-project.
 **Results

2.8. According to the JBI 2020 manual, authors are requested to present, as supplementary material, a table with excluded works and the reasons for exclusion.**

**Response:** Thank you for your comment. We added our data extraction form as supplementary material which refers to all excluded and included studies of initial screening, reference screening, and full-text screening. We refer to this with the following sentences in the section “Study Selection”:

*“Please refer to additional file S6 for an overview of excluded studies and the reason of
exclusion.”* (p. 13)

Moreover, we included a new Figure, which depicts the frequency of exclusion criteria.

***Figure 1*** *depicts the contribution of exclusion criteria.* (p. 13)

**Discussion

2.9. Authors are asked to present a topic/paragraph to address the strengths and limitations of this scoping review.**

**Response:** Thank you for your consideration. We now included a paragraph that address strengths and limitations of the present scoping review:

***Limitations***

*Although the scoping review was supported by steps including refinement of the protocol through team discussion, blinded searching, and selection of articles by two researchers, several limitations have to be mentioned. First, the present study did not include gray literature. Although the search included two relevant academic databases with peer-review journal articles, gray literature might also yield additional insights. Thus, it is possible that studies with human drone interaction focus have been overlooked. Second, the search protocol was based on a combination of keywords that may not capture all relevant studies. Third, the theoretical and practical implications derived from this scoping review need to be tested and validated with empirical research methods (e.g., MRC-Framework). Forth, we adopted a relatively narrow approach to the context by including supply of medicines and medical devices. Expanding the context might contribute to more hits according to human drone interaction settings. Nevertheless, the strength of this narrow context is to provide an insight in specific domains and its potential.* (p. 31)

**3. Minor issues

Introduction

3.1. Lines 71-77: citation #9 appears to be missing.**

**Response:** We now included the citation #9.

**3.2. I suggested that the authors justify/specify details about the originality of the review compared to the reviews already published.**

**Response:** Thank you for your suggestion. We specify details about the originality of the scoping review in the Introduction section. We added a paragraph that reads as following:

*„To date, reviews often focus on how drones are used for healthcare and its potentials [11–13]. For example, Hievert et al. [12] identified eight healthcare and health-related applications for drones. Drone delivered medical supplies and treatments was the most common identified application followed by environmental monitoring and using drones to deliver automated defibrillators (AEDs) indicating the importance of drones in improving access to health services. The review found out that all studies on drones are at a pilot stage that have not been implemented or adopted in healthcare settings. The identified studies focused on flight path and payload regulations, but there was limited attention to how patients and communities interact with drones. Rejeb et al. [13] identified three main barriers to humanitarian drones in technological, organizational, and environmental (regulatory) factors, called TOE barriers. Among other things, user acceptance is one organizational barrier to drone adoption in humanitarian logistics. The authors recommend applying technology adoption theories as well as using case studies, interviews, or surveys to understand success and failure factors of using drones within humanitarian organizations.*

*Hence, analysis of human drone interaction is required for understanding users’ needs and requirements in dealing and interaction with drones. The role of users, the involvement of users in drone delivery process and the handover of medical devices or urgently needed medicines are important factors in investigating and improving user acceptance. In the end, it is the user who will have to use the drone in everyday life and get along with it. Non-user-centered technologies will fail to be implemented as long as usefulness and usability are not a focus of research. As long as this is the case, people´s fears and concerns about drones will be the main obstacles to implementation although drones have a great potential in medical care and supply.*

*Within this healthcare supply context, the present paper is interested in identifying human drone interaction in real life scenarios that have received less attention in the ongoing debate. More specifically, the objective is to assess how human drone interaction in medical transport of medicines and devices in experimental studies is discussed in the academic literature.”* (p. 5-6)

**Methods

3.3. Lines 139-154: if the authors have used a template tool for data extraction, please include it in the additional information section.**

**Response:** We added our data extraction form as supplementary material which refers to all excluded and included studies of initial screening, reference screening, and full-text screening. We refer to this in the section “Study Selection”.

**Discussion

3.4. Lines 345-346: authors must insert reference(s) to support the statement presented in the discussion.**

**Response:** Thank you for your comment. We included the missing reference.

**3.5. Lines 348-357: authors must insert reference(s) to support the statement presented in the discussion.**

**Response:** Thank you. We now included the missing reference.

**3.6. Line 370: please inform which are the three studies.**

**Response:** We included the missing references.

**3.7. It is suggested that the authors discuss the results further in the context of the previous reviews identified.**

**Response:** Thank you very much for your helpful comment. We now discussed our results with a more narrow regard to previous reviews and theories. Please refer to the whole Discussion section for a complete view. We also integrated a further table (Table 4) which shows a summary of user experience, user feedbacks, and practical implication in the Result section and discussed them in the Discussion section.

**Table 4.** User experience, feedback, and practical implications.

|  | **problems** | **users´ concerns** | **users´ perceived support** | **suggestions of users** | **advantages from the researchers´ point of view** | **practical implications** |
| --- | --- | --- | --- | --- | --- | --- |
| (1) process / landing |  | uncertainty of arrival time [19,20] |  | noise emitting or something that alerts drone arrival [20] |  | test flights at day and night, using headlights, warning sensors, reflective tapes or sound |
|  |  | flight at night [20] |  | reflective tapes or lights for marking location of arriving [20] | in case of landing: warning sensors, lights, and sound to attract attention [17] |  |
|  |  | direct physical contact with drone: drone too close or propeller starts when approaching drone [18–20] |  | upward inclined or retractable propeller [18] | use a winch to avoid contact with drone [18] | release with winch over landing |
|  |  | landing in crowd areas [20] |  |  |  |  |
|  |  | issues with bystanders [20] |  |  |  |  |
|  |  | poor weather [20] |  |  |  | investigating complementary and interoperability of drones with other technologies |
|  |  | drone´s ability to find right location [20] |  |  |  | using applications with GPS tracking |
|  | using mobile phones [18] |  |  |  |  | educational training |
| (2) handover |  | finding AED fast enough [18,20] | drone´s hovering to find right location [18] | headlights in order to mark location of release [18] | live video streaming for facilitation of dispatcher orientation [18] | documentation of handover via live video streaming or app-based confirmation |
|  |  |  | red AED bag for easier identification on ground [18,20] |  |  |  |
|  | difficulty in removing AED from drone [20]  and  difficulties in handling AED despite dispatcher support [18] |  |  | colored Velcro tapes and additional labels with removing instructions [20] | suggestion of more intuitive location, e.g., on the top of drone [17] | using applications with instructions, e.g. via video |
| (3) communication |  | anxiety / uncertainty about direction or timing of drone´s arrival/landing [19,20] | interaction with dispatcher gave sense of security [18] | clearer instructions from dispatcher about appearance, look and sound of drone, direction, and landing [20] |  | clear and short instructions, education of professionals (e.g., dispatcher, pharmacists) |
|  | long sentences affect situation in negative way [18] |  |  |  | short encouraging sentences  observed positive  effect on compressions [18] |  |

**References

3.8. Some references are incomplete (eg, 21, 22, 23, 25, 26, 28, 29) and in disagreement with the journal's guidelines.**

**Response:** Thank you very much. We completed and updated the references according to the journal´s guidelines.
